# Goodbye to CFG

**DOI:** 10.1002/cfg.495

**Published:** 2005


					Comparative and Functional Genomics
Comp Funct Genom 2005; 6: 344.
Published online in Wiley InterScience (www.interscience.wiley.com). DOI: 10.1002/cfg.495
Editorial
Goodbye to CFG
Sadly, I am writing this Editorial to let you know of
our decision to close Comparative and Functional
Genomics as of this issue.
Unfortunately, despite much hard work by the
entire Editorial Board and the team at Wiley, CFG
has not achieved what we had hoped and we feel
that no amount of effort will be enough to rectify
this. This will be the final issue of the journal.
However, its archive will be maintained and all
the material published in CFG will continue to be
made available from the Journal's website.
I would like to take this opportunity to thank the
Section Editors and Editorial Board members, on
behalf of Wiley and myself, for all their work and
support for CFG.
I hope that you have enjoyed reading CFG and
send our thanks to all of you who contributed
articles to the journal or assisted in the refereeing
process.
Steve Oliver
Copyright  2006 John Wiley & Sons, Ltd.

				

